# Structure determination of amyrin isomers in cuticular waxes: a combined DFT/vibrational spectroscopy methodology†

**DOI:** 10.1039/d0ra00284d

**Published:** 2020-02-19

**Authors:** Luz D. M. Gómez-Pulido, Rafael C. González-Cano, Eva Domínguez, Antonio Heredia

**Affiliations:** IHSM La Mayora, Departamento de Mejora Genética y Biotecnología, Consejo Superior de Investigaciones Científicas E-29750 Algarrobo-Costa Málaga Spain heredia@uma.es +34-952131940; Departamento de Química Física, Facultad de Ciencias, Universidad de Málaga Málaga E-29071 Spain rafacano@uma.es; IHSM La Mayora, Departamento de Biología Molecular y Bioquímica, Universidad de Málaga E-29071 Málaga Spain

## Abstract

We present a new methodology for the structural characterization of amyrins, a class of triterpenoids found within the fruit and leaf cuticles of higher plants. Two amyrin isomers (α and β) have been studied taking into consideration a hydrophobic molecular scenario that mimics the cuticle matrix. DFT calculations have been employed in combination with experimental data from Raman vibrational spectroscopy and X-ray diffraction.

## Abstract 2

A new methodology for the structural characterization of amyrins based on DFT calculations and Raman vibrational spectroscopy.

## Graphical abstract

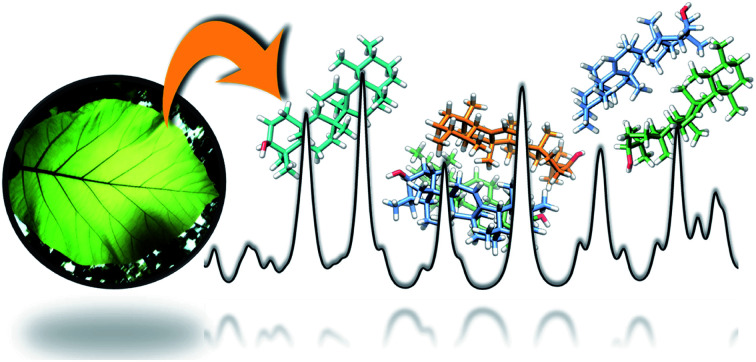

## 1. Introduction

The plant cuticle is a ubiquitous lipid membrane that covers the surface of aerial parts of higher plants. Its main functions are to regulate water and gas exchange with the environment, attenuate UV radiation and provide mechanical support. The main cuticle components are of lipid nature: the cutin matrix, a polyester made from interesterified polyhydroxy fatty acids, and waxes, which can be located on the outer surface of the cuticle or within the cutin matrix.^1^ Cuticle waxes are a complex mixture of very long chain alkanes, alcohols, fatty acids and triterpenoids.^1^ Triterpenoids are biologically versatile molecules formed from pentacyclic structures with recognized pharmaceutical and medical properties.^2^ Additionally, they have been shown to act as nanofillers, thanks to their intracuticular location,^2^ reducing the mobility of the cutin chains and thus conferring mechanical strength to the cuticle,^3^ in the same fashion as cuticle flavonoids.^4^

**Fig. 1:**
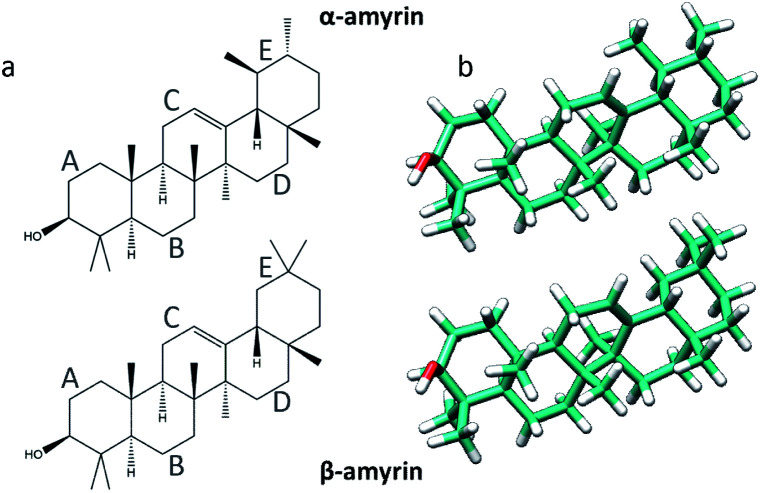
Representation of the α- (top) and β-amyrin (bottom) isomers on its schematic (a) and geometry optimized (b) structures.

Amyrin isomers are triterpenoids found in most fruit and leaf cuticles, being the α and β isomers (Fig. 1a) the main derivatives found in cuticular waxes.^2^ Methods and techniques as GC-MS, X-ray diffraction, calorimetry and FT-IR and Raman spectroscopy has been used to analyse and characterize the structure of these molecules^5,6^ but studies on the interactions of these molecules in the supramolecular arrangement into the cuticle scenario are still missing. Given their location filling the gaps within the cutin matrix, and the postulated clustering of cuticle flavonoids,^7^ it is most relevant to study the potential aggregation of these molecules. This will allow the discernment of the structure–property relationship of amyrin molecules, and provide insight into their role and distribution in the plant cuticle.

In order to develop a complete structural and molecular analysis for the α- and β-amyrin isomers, theoretical calculations have been carried out using the Density Functional Theory (DFT) method. Results are accompanied with the corresponding experimental Raman spectra and additional experimental data of these molecules. The structures were optimized within different environments using the Polarizable Continuum Model (PCM). This method uses the dielectric constant (*ε*) to emulate a given environment. In this case, three scenarios were analysed: isolated gas phase, *n*-octanol to mimic the average polarity present in the cutin matrix,^8^ and *n*-hexane for a more apolar, wax-clustered environment.

## 2. Methodology

### 2.1. Computational details

DFT calculations have been performed with Gaussian 16 software^9^ using the B3LYP functional together with the 6-31G** basis set. This is a hybrid functional combining the Hartree–Fock and Becke exact exchange functionals^10,11^ with the Lee–Yang–Parr correlation functional (LYP).^12^ It has been widely employed in geometric optimizations and in the evaluation of vibration frequencies. An empirical dispersion correction GD3 was used for the analysis of long-range intermolecular interactions.^13^ Theoretical Raman spectra were constructed after calculation of the vibrational normal modes using a FWHM (Full Width at Half Maximum) of 10 cm^−1^. Calculations were carried out in the Supercomputing and Bioinnovation Center (SCBI) of the University of Málaga.

The graphic editing of the optimized structures was done with the Chimera 1.11.2 software.^14^ Measurement of intermolecular distances between different monomer units was performed with Mercury 3.9.^15,16^ Experimental X-ray data were obtained from the ‘Cambridge Structural Database’ (CSD) webpage in order to compare between experimental and theoretical structural data.^17^ The experimental intermolecular distances between crystal unit cells have been obtained from the CIF files of the α- and β-amyrin derivatives (3β-acetoxy-α-amyrin^18^ and eπ–β-amyrin^19^).

### 2.2. Raman spectroscopy

Raman spectra were recorded with a Bruker Senterra Dispersive Raman microscope equipped with a Neon lamp and using a Nd:YAG laser with excitation at *λ* = 785 nm and 10 mW power at sample point. The microscope is coupled with a CCD 1 × 1 camera thermoelectrically cooled to −50 °C. A 40× magnification objective (Olympus) was employed and the laser spot size on sample was 1 μm. Each Raman spectrum was the average of 20 scans (4 accumulations of 10 seconds each) with a 3–5 cm^−1^ resolution. Sample integrity was checked after each measurement and spectra were registered at different temperatures, from 300 K to liquid N_2_ temperature, using a Linkam variable temperature sample cell. Samples were measured directly in the holder, in bulk and without previous preparation. Baseline correction was performed with OPUS 6.5 software.

## 3. Results and discussion

### 3.1. Analysis of the optimized molecular structures

The molecular structures of α- and β-amyrin were optimized under different polarity scenarios. The molecular backbone presents a deep distortion on the E ring in both isomers (Fig. 1b). The A ring, which holds the hydroxyl group, is the only cyclohexane that adopts a boat conformation. Because of this, the –OH group presents an axial configuration from the backbone plane. The C ring is a cyclohexene derivative whose double bond causes a flattening effect on the backbone. These conformational aspects may impact the ability of these molecules to aggregate. On the other hand, the different polarity environments herein studied did not cause significant differences in structure or energy of the optimized molecules (Fig. S1†).

**Fig. 2:**
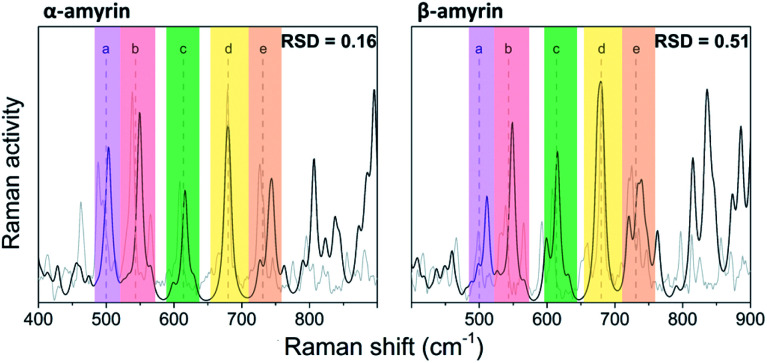
Normalized Raman spectra calculated for α- and β-amyrin in *n*-octanol (black) in comparison with the corresponding experimental spectrum determined at room temperature (gray).

The vibrational normal modes were also calculated in order to obtain a theoretical Raman spectrum for each isomer. The 400–900 cm^−1^ region was selected for the study since it includes the five bands associated with the C–C–C scissoring vibrational normal mode of the carbon atoms allocated in the different hexane rings of the amyrin backbone. These bands, highlighted in Fig. 2, correspond to the peaks: *a* ∼ 500 cm^−1^, *b* ∼ 540 cm^−1^, *c* ∼ 630 cm^−1^, *d* ∼ 680 cm^−1^, and *e* ∼ 740 cm^−1^. Their detailed normal modes can be found in Table TS1† and have been confirmed after Turner *et al.*^5^ Also, their corresponding eigenvectors are shown in Fig. S2.†

In order to compare the theoretical spectrum with the experimental one obtained at room temperature (300 K), the 400–900 cm^−1^ region was normalized with band *d*, since it showed the highest intensity. Fig. 2 presents the normalized calculated Raman spectra for α- and β-amyrin in *n*-octanol as well as the experimental one. The normalized spectra for the other environments studied are presented in Fig. S3.† Two relative intensity patterns were then constructed for the theoretical and the experimental Raman spectra of each isomer (Fig. S4†). These relative intensity patterns exhibited the same trend for the theoretical and experimental spectra in both isomers. Calculation of the relative standard deviation (RSD) for the theoretical spectrum of both isomers, using their corresponding experimental one as reference, allows quantifying the degree of similarity between spectra. Thus, variation between the relative activity of the calculated Raman and the relative intensity of the empirical spectrum for each band could be evaluated.^20^Table 1 presents the RSD average values of both isomers in the *n*-octanol environment. Results for the other polarity scenarios are included in Table TS2.† Comparison of the different scenarios showed that *n*-octanol gave the best results. However, the RSD values suggest that the monomeric molecule does not seem to be the best model for the isomer-pure amyrin framework.

**Table 1:** Relative Standard Deviation (RSD) values of the relative intensities for the calculated Raman spectra in *n*-octanol, RSD values of the intermolecular distances for all the proposed aggregates in comparison with the structures extracted from CSD in *n*-octanol, and relative binding energies (RBE) in kcal mol^−1^ for all the proposed aggregated structures of α- and β-amyrin in *n*-octanol

		Monomer	Dimer						Trimer					
			dim1	dim2	dim3	dim4	dim5	dim6	trim31	trim32	trim51	trim52	trim61	trim62
α-Amyrin	Raman RSD^a^	0.16	0.08	0.10	0.07	0.27	0.14	0.10	0.14	0.25	0.14	0.17	0.06	0.11
	Distance RSD^b^	—	0.87	0.48	0.18	0.15	0.24	0.16	0.31	0.09	0.32	0.17	0.30	0.07
	RBE (kcal mol^−1^)^c^	—	−5.9	−4.3	−7.2	−5.9	−8.7	−8.2	−9.1	−11.2	−10.3	−13.8	−9.5	−12.0
β-Amyrin	Raman RSD^a^	0.51	0.50	0.42	0.43	0.54	0.27	0.25	0.41	0.45	0.44	0.31	0.33	0.48
	Distance RSD^b^	—	0.61	0.41	0.11	0.12	0.01	0.07	0.32	0.08	0.28	0.06	0.11	0.07
	RBE (kcal mol^−1^)^c^	—	−6.2	−5.6	−7.3	−6.1	−6.4	−7.4	−9.2	−13.4	−11.0	−12.8	−13.5	−13.5

a Averaged from the Raman bands relative intensity RSD.

b Averaged from the intermolecular distance RSD.

c Obtained from the BE calculated per amyrin unit in the cell.

### 3.2. Analysis of dimeric aggregated structures

**Fig. 3:**
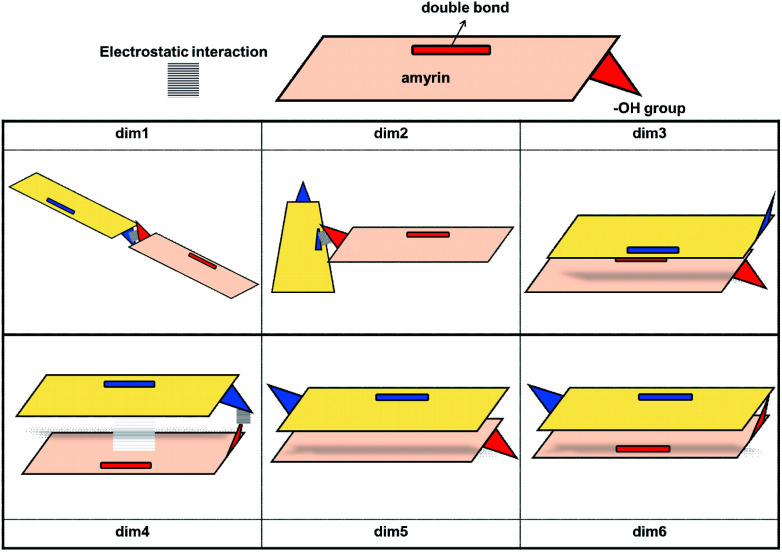
Schematic representation of the proposed dimeric aggregates.

Given the above-mentioned results, the possibility of homodimer aggregation of α and β amyrins were analysed. Six possible conformations have been studied, named ‘dim1’ to ‘dim6’ (Fig. 3). Three of them are linked by electrostatic forces between the hydroxyl group and the double bond (hydrogen bonds between hydroxyl groups, dim1; π–π interaction, dim2; hydroxyl–π interaction, dim4). The other three structures represent different overlapping molecules based on the orientation of their hydroxyl groups with no specific attraction force (antiparallel backbones with faced, dim3, and opposite, dim5, hydroxyl groups and parallel backbones with hydroxyl groups in the opposite position, dim6). All the dimer structures have been optimized in the three polarity environments and analysed according to their structure, aggregation energy and vibrational spectra data.

The RSD for the intermolecular distances, using the corresponding experimental data as reference, are included in Table 1. Analysis of the results allows the conclusion that the arrangement of dim1 and dim2, both dimers with an electrostatic attraction, are far from the experimental data for both α- and β-amyrin. This implies that, regarding the distance RSD analysis, dimer aggregation is mainly ruled by distance-dependent interaction (dim3, dim5 and dim6) although dim4, having electrostatic interaction, cannot be ruled out. The intermolecular distances for the proposed dimers in the two other environments are presented in Table TS3.† These results barely show any difference in *n*-hexane or with no PCM correction compared to *n*-octanol, for both α- and β-amyrin. Thus, it can be inferred that polarity will only have a slight effect on the relative position of the molecules.

The stability analysis was afforded with the calculation of the Binding Energy (BE), in order to preview the most probable dimeric aggregation taking into consideration only the energetic aspects (Table TS4†). The relative binding energy (RBE) allows to compare the stabilization of each molecule in the dimer with the monomeric one (Table 1). This analysis reveals that the dimeric aggregation in *n*-octanol tends to energetically favour dim3, dim5 or dim6 conformations; hence, the electrostatic interactions do not have any impact in the stabilization of α- or β-amyrin dimers.

A detailed analysis comparing the different polarity scenarios (Table TS4†) show small differences among them. Mainly, *n*-hexane and the isolated gas phase showed a more negative RBE than *n*-octanol for all the structures analysed. This implies better dimer stabilization when the polarity scenario changes from that representative of the cutin matrix to waxes. Amyrin molecules could be slightly more unstable in these apolar environments and consequently increase their tendency to aggregate.

The theoretical vibrational Raman spectra were calculated for all the proposed dimeric structures in the three PCM scenarios (Fig. S5†). Comparison with the experimental spectra was carried out by means of RSD calculation of their relative intensities (Fig. S6†), using the five selected bands previously mentioned. Table 1 shows the Raman RSD results in *n*-octanol. An improvement in the theoretical–experimental correlation can be observed in the dimers compared with the monomeric structure. An exception to this overall trend is dim4 for both amyrin isomers and dim1 for β-amyrin. The better fitting of dim3, dim5 and dim6 with the empirical Raman spectrum for both isomers again confirms the presence of conformers aggregated by distance–dependent interaction in the sample. Regarding the other polarity scenarios, the results were quite similar to that of *n*-octanol which, in turn, gave the best results. This suggests that *n*-octanol better represents the amyrin environment in the sample.

**Fig. 4:**
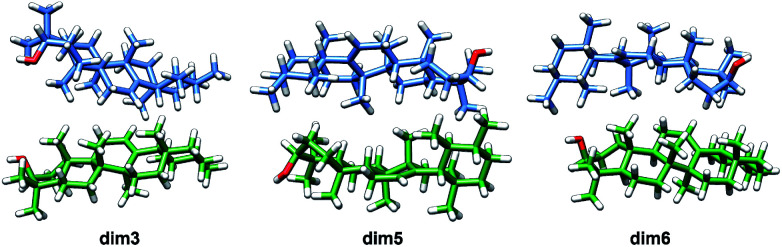
Optimized structures of the α-amyrin dimers aggregated by distance–dependent interaction.

Taking into consideration the above mentioned results, it can be then concluded that dimers aggregated by non-electrostatic interactions represent the best model for α- and β-amyrin homodimers (Fig. 4).

### 3.3. Analysis of trimeric aggregated structures

According to the literature, the reported amyrin derivatives^18,19^ show a crystalline arrangement based on a three molecules unit cell (CSD, Fig. S7†). Therefore, the analysis of the most stable amyrin crystal structure was focused on the trimeric aggregation unit cell.

**Fig. 5:**
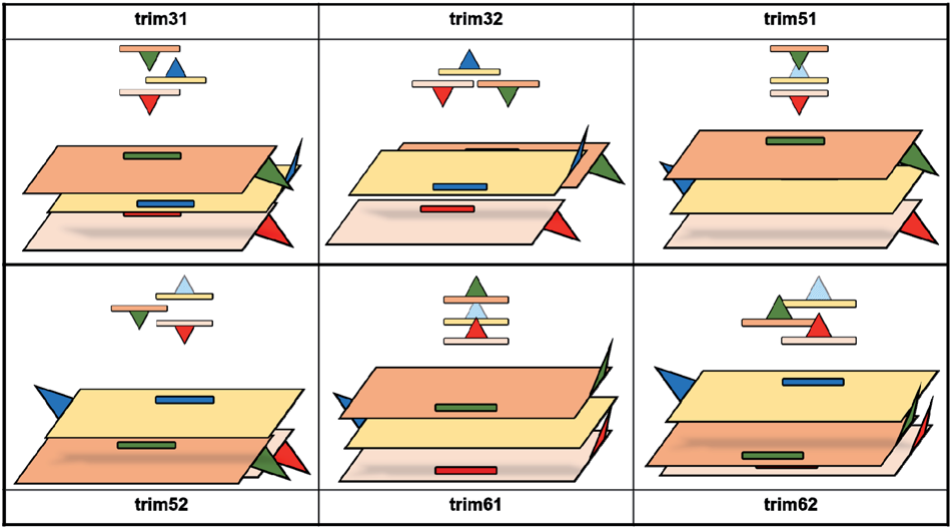
Schematic representation of the proposed trimeric aggregates. In red and blue the initial dimer, in green the additional amiryn monomer. Hydroxyl groups facing or extending away from the hydroxyl groups of the other amyrin molecules are represented in dark blue or dashed light blue, respectively.

Since the previous analysis of the dimeric structures indicated that aggregation is not dominated by π or hydroxyl electrostatic effects, the addition of a third amyrin molecule has only been analysed for dim3, dim5 and dim6 in two possible conformations: an overall block (trim31, trim51 and trim61) or trigonal arrangement (trim32, trim52 and trim62). A description and scheme of the proposed trimeric structures is shown in Fig. 5.

**Fig. 6:**
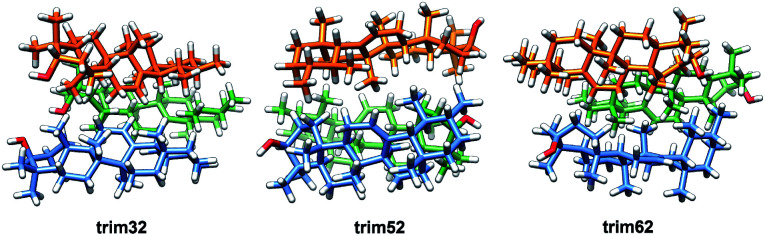
Optimized structures of the α-amyrin trigonal trimers.

In the crystalline structures of α- and β-amyrin derivatives, the intermolecular distances in the unit cell are approximately homogeneous^18,19^ (Table TS3†). This is in contrast with the calculated data for the in-block trimers (trim31, trim51 and trim61) for both isomers (except for trim61 in β-amyrin) where the distances were notably higher (Table 1). However, the intermolecular distances for trigonal trimers (trim32, trim52 and trim62) (Fig. 6) are in agreement with the reference values. As it has been pointed out earlier, the polarity scenario only had a minor effect on the position of the molecules in the optimized structures (Table TS3†).

The RBE analysis was also performed for the trimeric aggregates. The stabilization energy per amyrin unit with the addition of an additional unit to the dimer was calculated and the results obtained in *n*-octanol are presented in Table 1. Compared to the dimeric aggregation, formation of a trimeric cell provides an extra stabilization of the molecules, for both amyrin isomers. This confirms the tendency of amyrins to form trimeric aggregations. Comparison of the in-block construction with the trigonal arrangement, for α- and β-amyrin, indicated a higher stabilization of the trigonal structures (Table 1) [*e.g.*, the gain of stability for α-amyrin from dim5 to trim51/trim52 is −1.6/−5.1 kcal mol^−1^, and for β-amyrin from dim3 to trim31/trim32 is −1.9/−6.1 kcal mol^−1^]. This is also in agreement with the unit cell structure reported for these molecules in the CSD (Fig. S7†). Besides, the value of RBE between the trimeric conformers is very similar, which would allow them to interconvert their structures. The minor changes in energy stabilization with polarity, discussed in the previous section for dimer formation, are not present in the trimeric aggregations (Table TS4†). The comparison of dimer and trimer aggregates indicate that, although trimers showed better RBE values, these differences were small and dimers are already stable aggregates, regardless the environment analysed. It should be mentioned that, the identical RBE values obtained for trim61 and trim62 in β-amyrin were the result of the structural conversion of trim61 into its corresponding trigonal homolog caused by instability during the geometric optimization.

The theoretical Raman spectra for the proposed trimers have been simulated in the three possible scenarios (Fig. S8†) and compared with the experimental Raman data. Their relative intensity patterns can be found in Fig. S9.† The calculated RSD values for these spectra in *n*-octanol did not show an improvement compared with the dimers, indicating that the sample is not only populated by the most stable conformer but it probably presents a combination of the most stable structures (Table 1). Again, there is a better consonance between the experimental and the calculated spectra in *n*-octanol compared with the other environments. For this reason, further analyses will only be carried out in this scenario.

### 3.4. Analysis of amyrin crystalline growth

**Fig. 7:**
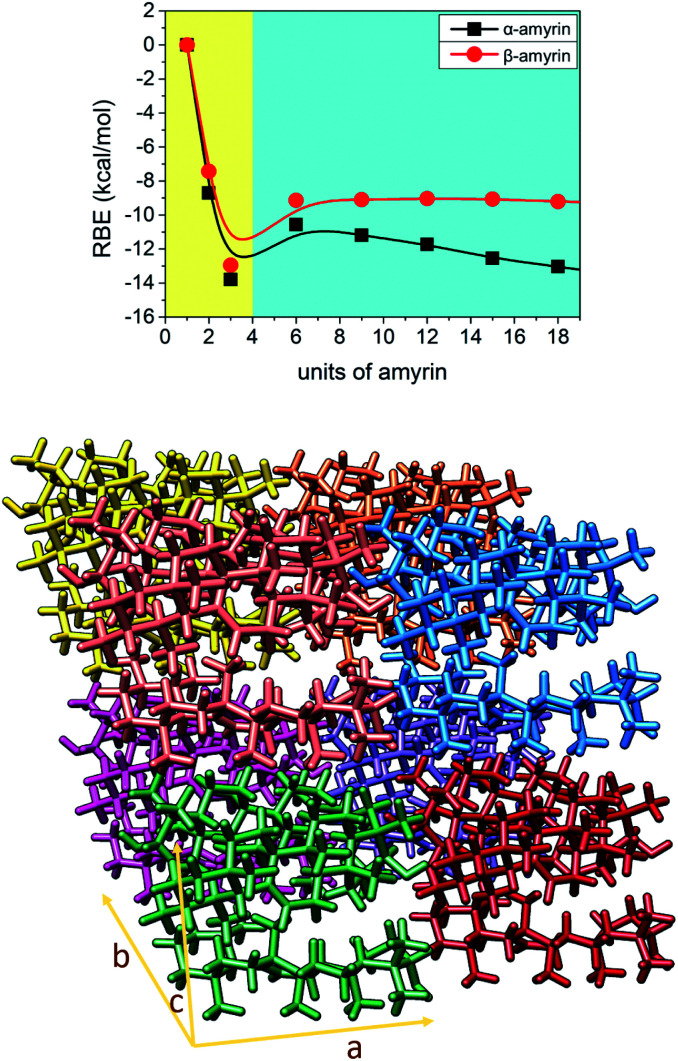
Representation of RBE *vs.* number of α- and β-amyrin units considering a crystalline growth. The plot has been divided in two areas: a quick energetic stabilization (left, in yellow) and a moderated arranged stabilization (right, in blue). At bottom: representation of a 2 × 2 × 2 crystalline cell of α-amyrin with a trim52 unit cell arrangement.

Given the above mentioned results, the ability of crystalline growth has been analysed for both amyrin isomers, considering the aggregation of trigonal trimeric cells. RBE calculation for different crystal sizes from 1 to 18 molecular units is shown in Fig. 7 together with a model of amyrin crystalline structure based on these unit cells. This analysis allows the study of crystalline growth and stabilization. Two types of growth can be observed, one based on the formation of the trimeric unit cell (highlighted in yellow) and a second one based on the incorporation of additional unit cells (highlighted in blue). The first type of growth is accompanied by a significant stabilization per molecular unit (from monomer to dimer and trimer), specifically an energy of stabilization up to 14 kcal mol^−1^ per amyrin unit. After the formation of the growing unit cell, an immediate loss of stabilization is observed after the addition of another cell. This is followed by a stable RBE with crystal growth for β-amyrin and a slight increase in stability for α-amyrin. To summarise, the crystalline growth of amyrin is energetically less favourable after the formation of the trimer. Thus, amyrin is expected to have low crystallinity. This could explain the description of amyrin structure as a set of independent crystalline clusters (semicrystalline arrangement) reported in the recent literature.^6^ Moreover, these results are in agreement with the low crystallinity attributed to triterpenoids in the cuticle of *Diospiros kaki*.^3^

### 3.5. Aggregated structures conformational distribution

As it has been already pointed out, amyrins have the ability of conformational interconversion and, thus, they will present a conformational distribution based on a Maxwell–Boltzmann population. To recreate the Raman spectrum of this Maxwell–Boltzmann theoretical approach, it is previously necessary to weight the spectrum for each dimeric and trimeric conformer.^21,22^ The following premise will be taken into account:1
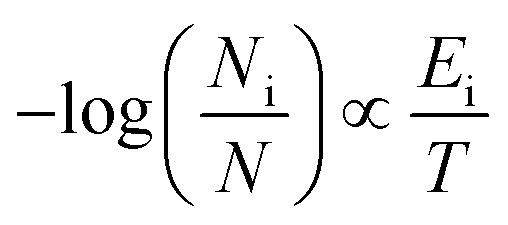
where *N*_i_ is the expected account of particles within a given microstate, *N* is the total number of particles within the system, *E*_i_ is the energy that characterizes each of the microstate and *T* is the temperature of the system in a state of equilibrium.

**Fig. 8:**
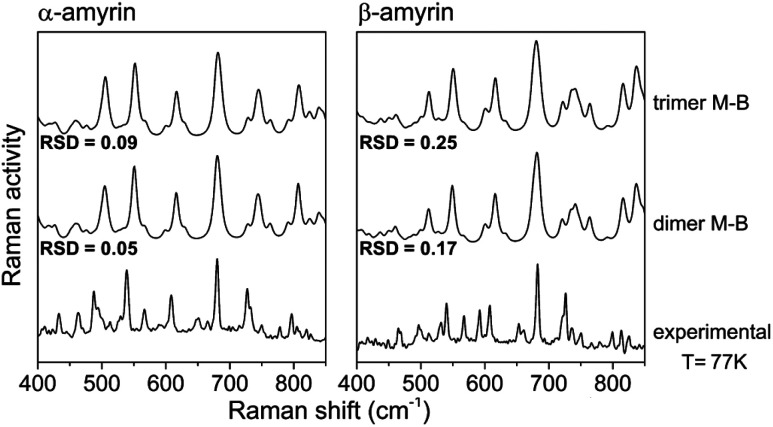
Maxwell–Boltzmann weighted calculated Raman spectra for the α- and β-amyrin dimers and trimers in *n*-octanol together with the experimental Raman spectrum at 77 K for each isomer.

Fig. 8 shows an average of the theoretical Raman spectra obtained by summing the weighted individual spectra of the dimeric and trimeric conformers according to their relative Maxwell–Boltzmann populations (M–B) as deduced by (1). For this purpose, only the distance–dependent dimers and trimers have been selected, that is, dim3, dim5 and dim6 (dimer M–B) and all the analysed trimers (trimer M–B). The relative concentration of each conformer is plotted in Fig. S10.†

Table TS5† shows the RSD values for dimers and trimers as well as their corresponding M–B weighted spectra, taking the Raman spectra at different temperatures, from *T* = 300 K to liquid N_2_ temperature (*T* = 77 K), as reference. Chiefly, RSD values of the M–B weighted spectra present a better fitting with the experimental Raman spectra at any temperature, more notably at lower ones. This demonstrate that the model of a conformational blend of amyrin structures better describes the analysed experimental sample. Table 2 presents the RSD calculation of the M–B dimers and trimers, taking the Raman spectra at different temperatures as reference. As the temperature decreases, a better consonance between the M–B calculated and the empirical spectra is found as consequence of the loss of thermal energy in the system that inhibits conformer interconversion^21,23^

**Table 2:** Relative standard deviation (RSD) values for the calculated Raman spectra in *n*-octanol averaged by Maxwell–Boltzmann distribution (M–B) and taking the experimental data at different temperatures as reference

Aggregation	α-Amyrin				β-Amyrin			
	300 K	200 K	150 K	77 K	300 K	200 K	150 K	77 K
M–B dimers	0.09	0.08	0.06	0.05	0.26	0.18	0.20	0.17
M–B trimers	0.11	0.11	0.10	0.09	0.35	0.26	0.28	0.25

To sum up, there is a good agreement between the theoretical model presented as a M–B distribution and the isomeric-pure sample of amyrin. Thus, the system can be described as a group of different arranged aggregations that forms a semicrystalline framework.

## 4. Conclusions

A methodology to determine the structural arrangement of low crystallinity molecules, such as α- and β-amyrin, has been developed. The study compiles three different types of analyses: stabilization energy, structural arrangement and spectroscopy.

Stabilization energy and structural analysis confirms a major tendency of amyrins to form trimeric trigonal aggregates with a semicrystalline arrangement. Spectroscopy analyses indicate that amyrin molecules are present as a blend of the most stable conformers, that is, dimers aggregated by distance–dependent interactions and their corresponding derived trigonal trimers.

These results provide valuable information on the arrangement of amyrin molecules in an *in vitro* scenario that will allow a good approximation to the functionality and structure of these triterpenoids in future work. From a biological perspective, this is a first approach to the study of the location and distribution of amyrin molecules within the cuticle.

## Conflicts of interest

There are no conflicts to declare.

## Supplementary Material

RA-010-D0RA00284D-s001
