# Supplementary material for: Structure determination of amyrin isomers in cuticular waxes: a combined DFT/vibrational spectroscopy methodology
Source: RSC Adv. 2020 Feb 19;10(13):7654–60. doi: 10.1039/d0ra00284d (PMC9049878; doi:10.1039/d0ra00284d)
Supplement: RA-010-D0RA00284D-s001 [file RA-010-D0RA00284D-s001.pdf]

Electronic Supplementary Material (ESI) for RSC Advances.
This journal is © The Royal Society of Chemistry 2020

Electronic Supplementary Information

Figure S1.- Representation of the α- and β-amyrin isomers on their optimized forms with no PCM correction (left) and in *n*-hexane (right)

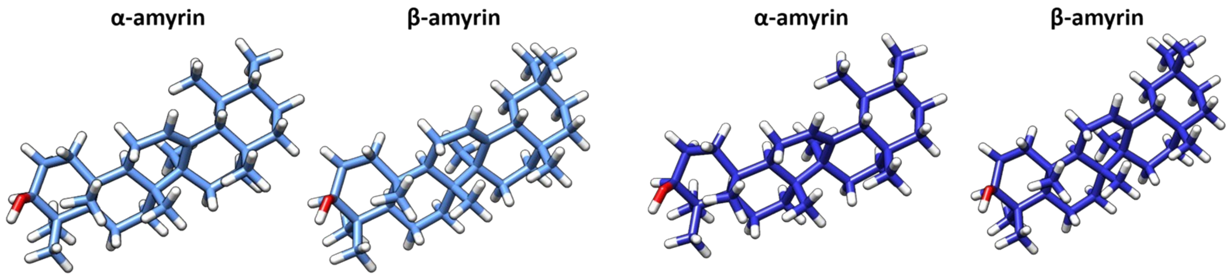

Table TS1.- Vibrational normal modes analysed in the Raman spectra

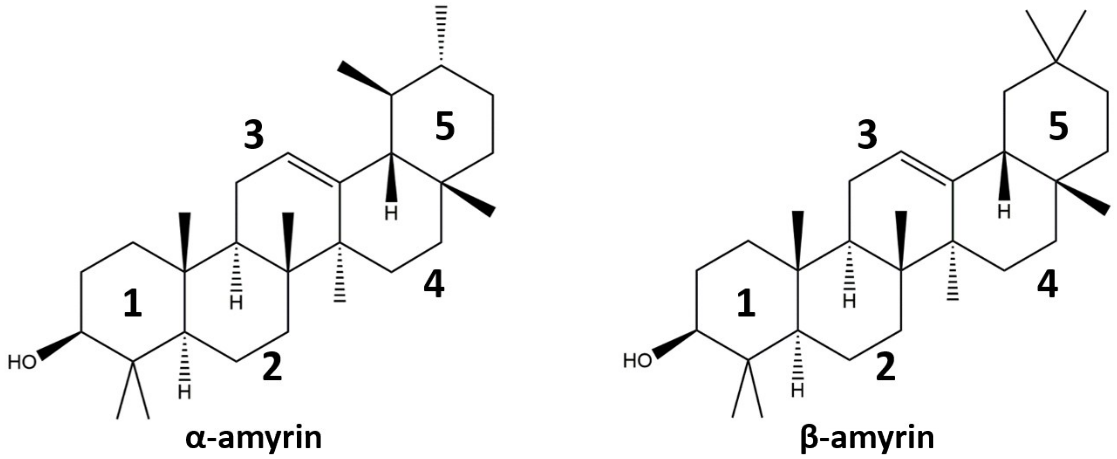

Band	Raman shift	Description	Symbol
a	~750 cm^{-1}	CCC scissoring at rings 4 and 5	$\delta(CCC)_{45}$
b	~680 cm^{-1}	CCC scissoring at ring 3	$\delta(CCC)_{3}$
c	~630 cm^{-1}	CCC scissoringg at ring 4	$\delta(CCC)_{4}$
d	~550 cm^{-1}	CCC scissoring at rings 1 and 2	$\delta(CCC)_{12}$
e	~500 cm^{-1}	CCC scissoring at ring 5	$\delta(CCC)_{5}$

Figure S2.- Representation of the eigenvectors calculated for each of the five selected bands chosen in the Raman spectra.

α-amyrin

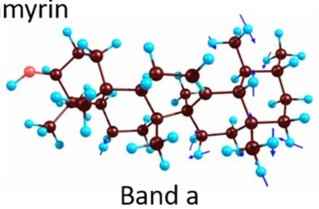

Band a

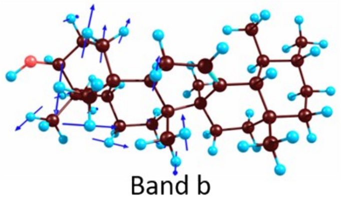

Band b

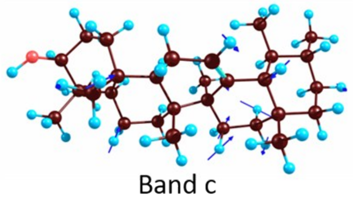

Band c

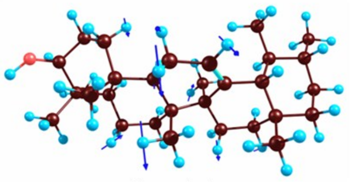

Band d

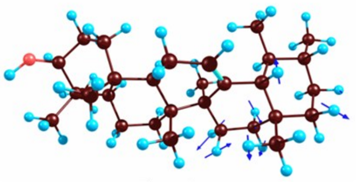

Band e

β-amyrin

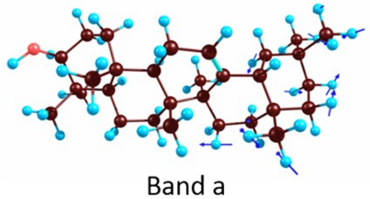

Band a

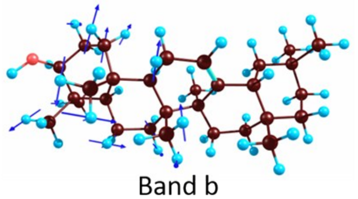

Band b

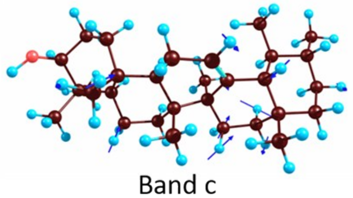

Band c

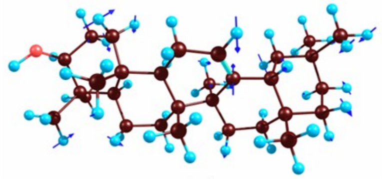

Band d

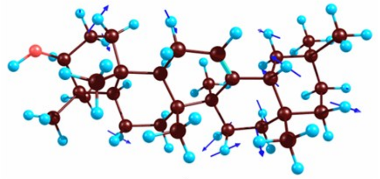

Band e

Figure S3- Theoretical Raman spectra (black) calculated for α- and β-amyrin with no PCM correction and in *n*-hexane. Their corresponding experimental spectra (gray) determined at room temperature are also shown.

NO PCM CORRECTION

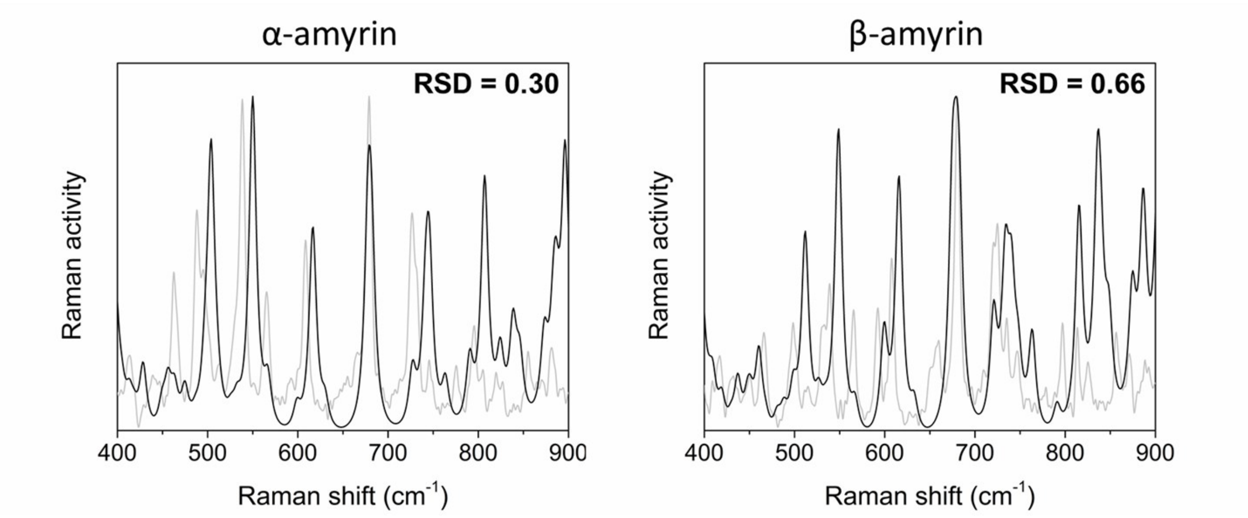

n-HEXANE

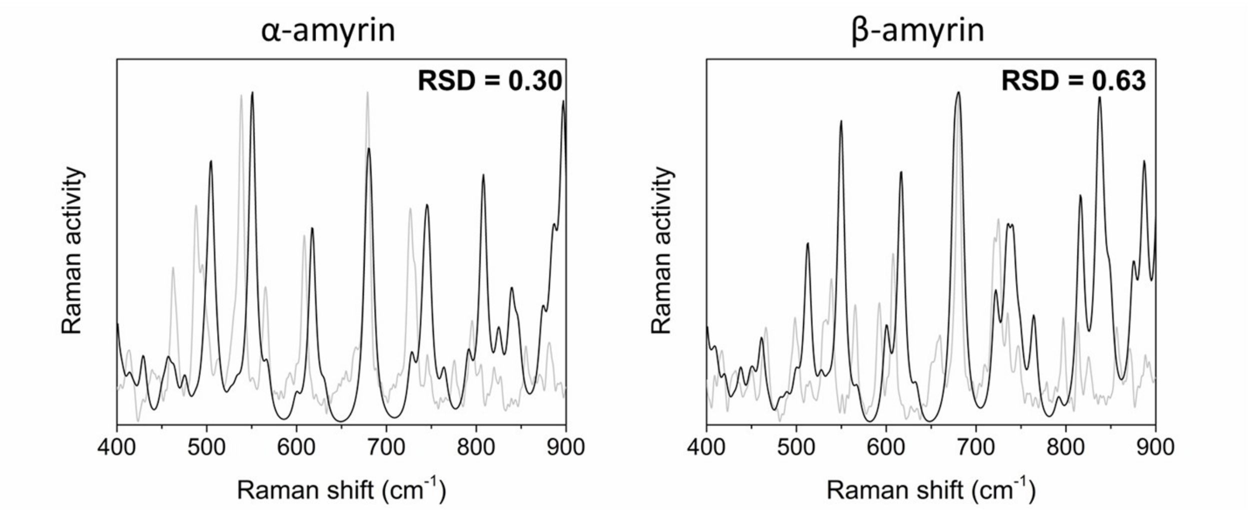

Figure S4.- Relative intensity patterns of the experimental (square) and the theoretical Raman spectra for α- and β-amyrin. The three theoretical environments studied were *n*-octanol (upside triangle), *n*-hexane (downside triangle) and no PCM correction (circle).

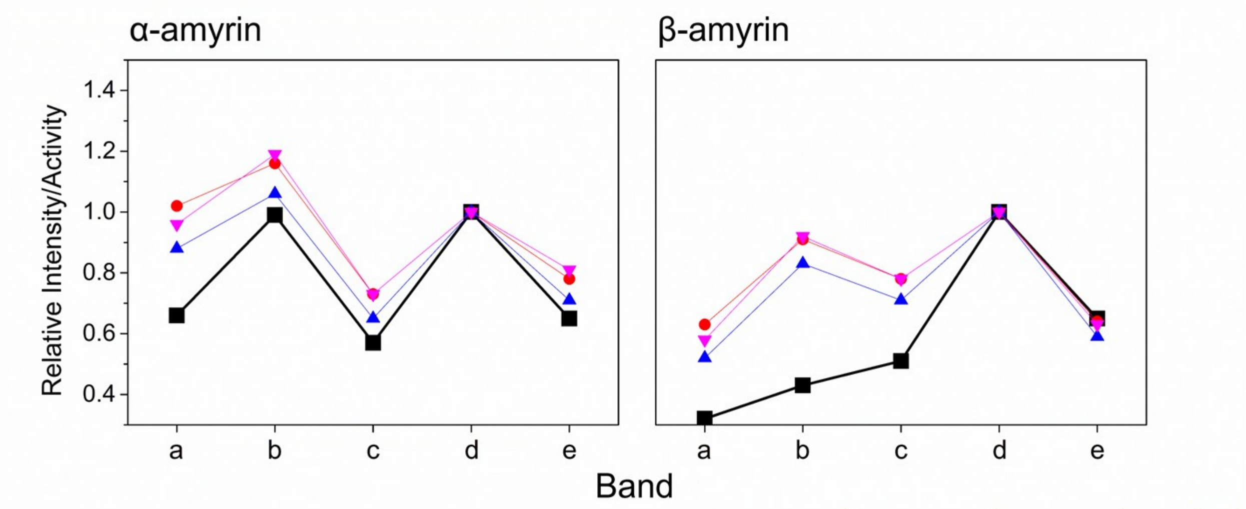

Table TS2.- Relative Standard Deviation (RSD) values for the calculated Raman spectra in the three PCM options for all the structures analysed.

NO PCM CORRECTION

	Band	Monomer	Dimer						Trimer					
			dim1	dim2	dim3	dim4	dim5	dim6	trim31	trim32	trim51	trim52	trim61	trim62
α-amyrin	a	0.55	0.33	0.24	0.21	0.52	0.00	0.35	0.09	0.56	0.17	0.20	0.17	0.55
	b	0.17	0.09	0.05	0.24	0.44	0.26	0.01	0.15	0.44	0.29	0.17	0.01	0.16
	c	0.28	0.40	0.30	0.21	0.37	0.09	0.05	0.19	0.28	0.19	0.07	0.05	0.12
	e	0.20	0.15	0.09	0.08	0.55	0.25	0.12	0.20	0.18	0.29	0.26	0.06	0.00
	average	**0.30**	**0.25**	**0.17**	**0.19**	**0.47**	**0.15**	**0.13**	**0.16**	**0.37**	**0.24**	**0.18**	**0.07**	**0.21**
β-amyrin	a	0.97	0.66	0.53	0.56	0.91	0.47	0.69	0.28	0.75	0.47	0.25	0.75	0.59
	b	1.12	0.77	0.79	1.16	1.37	0.63	1.02	0.91	0.91	1.12	0.44	0.79	0.93
	c	0.53	0.41	0.33	0.39	0.69	0.10	0.43	0.18	0.29	0.37	0.00	0.18	0.37
	e	0.02	0.78	0.17	0.11	0.06	0.22	0.15	0.32	0.05	0.11	0.38	0.08	0.08
	average	**0.66**	**0.66**	**0.46**	**0.56**	**0.76**	**0.35**	**0.57**	**0.42**	**0.50**	**0.52**	**0.27**	**0.45**	**0.49**

n-OCTANOL

	Band	Monomer	Dimer						Trimer					
			dim1	dim2	dim3	dim4	dim5	dim6	trim31	trim32	trim51	trim52	trim61	trim62
α-amyrin	a	0.33	0.20	0.20	0.06	0.32	0.08	0.09	0.20	0.33	0.05	0.00	0.15	0.35
	b	0.07	0.05	0.01	0.05	0.22	0.22	0.13	0.05	0.27	0.33	0.18	0.03	0.01
	c	0.14	0.07	0.16	0.12	0.19	0.05	0.04	0.07	0.25	0.16	0.16	0.07	0.07
	e	0.09	0.02	0.02	0.03	0.35	0.20	0.12	0.02	0.14	0.03	0.32	0.00	0.03
	average	**0.16**	**0.08**	**0.10**	**0.07**	**0.27**	**0.14**	**0.10**	**0.14**	**0.25**	**0.14**	**0.17**	**0.06**	**0.11**
β-amyrin	a	0.63	0.63	0.41	0.50	0.63	0.28	0.16	0.38	0.66	0.47	0.31	0.66	0.50
	b	0.93	0.91	0.74	0.81	1.02	0.40	0.42	0.81	0.77	0.70	0.44	0.49	0.91
	c	0.39	0.27	0.33	0.35	0.51	0.04	0.02	0.24	0.29	0.33	0.10	0.16	0.37
	e	0.09	0.18	0.18	0.06	0.00	0.35	0.40	0.23	0.08	0.25	0.40	0.00	0.14
	average	**0.51**	**0.50**	**0.42**	**0.43**	**0.54**	**0.27**	**0.25**	**0.41**	**0.45**	**0.44**	**0.31**	**0.33**	**0.48**

n-HEXANE

	Band	Monomer	Dimer						Trimer					
			dim1	dim2	dim3	dim4	dim5	dim6	trim31	trim32	trim51	trim52	trim61	trim62
α-amyrin	a	0.45	0.30	0.21	0.03	0.53	0.03	0.20	0.20	0.39	0.20	0.03	0.12	0.42
	b	0.20	0.06	0.01	0.14	0.44	0.22	0.10	0.14	0.33	0.25	0.05	0.01	0.08
	c	0.28	0.26	0.23	0.19	0.42	0.04	0.02	0.18	0.26	0.16	0.32	0.00	0.16
	e	0.25	0.12	0.09	0.00	0.49	0.14	0.09	0.18	0.11	0.26	0.03	0.02	0.02
	average	**0.30**	**0.19**	**0.14**	**0.09**	**0.47**	**0.11**	**0.19**	**0.17**	**0.27**	**0.22**	**0.11**	**0.04**	**0.17**
β-amyrin	a	0.81	0.74	0.53	0.53	0.72	0.47	0.28	0.25	0.69	0.78	0.22	0.81	0.56
	b	1.14	0.93	0.77	0.91	1.23	0.37	0.35	0.86	0.84	1.12	0.37	0.47	0.93
	c	0.53	0.27	0.45	0.39	0.61	0.04	0.06	0.18	0.29	0.43	0.08	0.20	0.35
	e	0.03	0.22	0.15	0.08	0.08	0.35	0.42	0.29	0.09	0.15	0.40	0.12	0.06
	average	**0.63**	**0.53**	**0.48**	**0.48**	**0.66**	**0.31**	**0.28**	**0.39**	**0.48**	**0.62**	**0.27**	**0.40**	**0.48**

Table TS3.- Comparison of the experimental and calculated intermolecular distances for all the aggregated structures studied (dimers and trimers) in the three PCM options. Each theoretical value is related to the empirical by the RSD.

NO PCM CORRECTION

Distance (Å)	Experimental	Dimer						Trimer					
		dim1	dim2	dim3	dim4	dim5	dim6	trim31	trim32	trim51	trim52	trim61	trim62
α-amyrin	7.7							6.6	6.6	6.0	6.0	6.4	6.7
	8.0	14.8	11.8	6.5	6.7	6.0	6.6	7.0	7.8	6.6	6.7	7.4	8.7
	8.0							13.4	8.3	12.5	6.8	13.3	9.5
	**RSD**	**0.87**	**0.49**	**0.18**	**0.15**	**0.24**	**0.16**	**0.31**	**0.07**	**0.32**	**0.18**	**0.30**	**0.13**
β-amyrin	7.5							6.6	6.6	6.9	6.9	6.4	6.3
	7.5	15.1	10.6	6.6	6.6	7.2	6.3	6.7	6.8	7.6	7.0	7.0	7.3
	7.5							13.0	7.4	9.4	7.2	7.7	7.7
	**RSD**	**1.01**	**0.41**	**0.12**	**0.12**	**0.04**	**0.16**	**0.32**	**0.08**	**0.12**	**0.06**	**0.08**	**0.07**

n-OCTANOL

Distance (Å)	Experimental	Dimer						Trimer					
		dim1	dim2	dim3	dim4	dim5	dim6	trim31	trim32	trim51	trim52	trim61	trim62
α-amyrin	7.7							6.6	6.5	6.0	6.0	6.5	6.5
	8.0	14.8	11.7	6.5	6.7	6.0	6.6	7.0	7.4	6.6	6.8	7.4	7.6
	8.0							13.4	8.4	12.6	6.8	13.3	7.9
	**RSD**	**0.87**	**0.48**	**0.18**	**0.15**	**0.24**	**0.16**	**0.31**	**0.09**	**0.32**	**0.17**	**0.30**	**0.07**
β-amyrin	7.5							6.7	6.6	7.4	6.9	6.3	6.3
	7.5	12.1	10.6	6.7	6.6	7.4	7.0	6.7	6.8	7.5	7.0	6.7	7.3
	7.5							13.0	7.4	13.6	7.2	8.0	7.7
	**RSD**	**0.61**	**0.41**	**0.11**	**0.12**	**0.01**	**0.07**	**0.32**	**0.08**	**0.28**	**0.06**	**0.11**	**0.07**

n-HEXANE

Distance (Å)	Experimental	Dimer						Trimer					
		dim1	dim2	dim3	dim4	dim5	dim6	trim31	trim32	trim51	trim52	trim61	trim62
α-amyrin	7.7							6.6	6.6	6.0	6.0	6.5	6.5
	8.0	14.8	11.6	6.5	6.7	6.0	6.6	7.0	7.8	6.6	6.7	7.4	7.6
	8.0							13.4	8.3	12.6	6.8	13.3	7.9
	**RSD**	**0.87**	**0.47**	**0.18**	**0.15**	**0.24**	**0.16**	**0.31**	**0.07**	**0.32**	**0.18**	**0.30**	**0.07**
β-amyrin	7.5							6.6	6.6	7.3	6.9	6.3	6.3
	7.5	12.0	10.6	6.6	6.6	7.4	7.0	6.7	6.8	7.6	7.0	6.7	7.3
	7.5							13.0	7.4	11.0	7.2	8.0	7.7
	**RSD**	**0.60**	**0.41**	**0.12**	**0.12**	**0.01**	**0.07**	**0.32**	**0.08**	**0.17**	**0.06**	**0.11**	**0.07**

Table TS4.- Binding energies (BE) and relative binding energies (RBE) in kcal·mol^{-1} for all the aggregated structures of α- and β-amyrin in the three PCM options.

NO PCM CORRECTION

Energy (kcal·mol^{-1})		Dimer						Trimer					
		dim1	dim2	dim3	dim4	dim5	dim6	trim31	trim32	trim51	trim52	trim61	trim62
α-amyrin	BE	-12.5	-9.4	-14.7	-11.8	-17.6	-16.9	-27.5	-34.5	-31.2	-42.2	-29.3	-36.1
	RBE	-6.2	-4.7	-7.3	-5.9	-8.8	-8.5	-9.2	-11.5	-10.4	-14.1	-9.8	-12.0
β-amyrin	BE	-10.9	-12.2	-14.9	-12.3	-13.1	-19.7	-27.5	-40.1	-33.0	-38.3	-40.6	-40.4
	RBE	-5.4	-6.14	-7.4	-6.2	-6.5	-9.9	-9.2	-13.4	-11.0	-12.8	-13.5	-13.5

n-OCTANOL

Energy (kcal·mol^{-1})		Dimer						Trimer					
		dim1	dim2	dim3	dim4	dim5	dim6	trim31	trim32	trim51	trim52	trim61	trim62
α-amyrin	BE	-11.8	-8.5	-14.5	-11.7	-17.4	-16.3	-27.2	-33.7	-30.8	-41.4	-28.5	-36.1
	RBE	-5.9	-4.3	-7.2	-5.9	-8.7	-8.2	-9.1	-11.2	-10.3	-13.8	-9.5	-12.0
β-amyrin	BE	-12.3	-11.1	-14.7	-12.3	-12.8	-14.9	-27.5	-40.1	-33.0	-38.3	-40.6	-40.4
	RBE	-6.2	-5.6	-7.3	-6.1	-6.4	-7.4	-9.2	-13.4	-11.0	-12.8	-13.5	-13.5

n-HEXANE

Energy (kcal·mol^{-1})		Dimer						Trimer					
		dim1	dim2	dim3	dim4	dim5	dim6	trim31	trim32	trim51	trim52	trim61	trim62
α-amyrin	BE	-12.1	-9.0	-14.6	-11.7	-17.5	-16.7	-27.3	-34.2	-31.0	-41.9	-29.0	-36.5
	RBE	-6.1	-4.5	-7.3	-5.9	-8.7	-8.3	-9.1	-11.4	-10.3	-14.0	-9.7	-12.2
β-amyrin	BE	-13.9	-12.9	-15.9	-13.4	-14.0	-16.2	-27.4	-39.6	-30.9	-38.1	-39.7	-40.0
	RBE	-6.9	-6.5	-7.9	-6.7	-7.0	-8.1	-9.1	-13.2	-10.3	-12.7	-13.2	-13.3

Figure S5.- Comparison of the experimental Raman spectra at room temperature (gray) with those calculated for α- and β-amyrin dimers in the three PCM options (black).

NO PCM CORRECTION

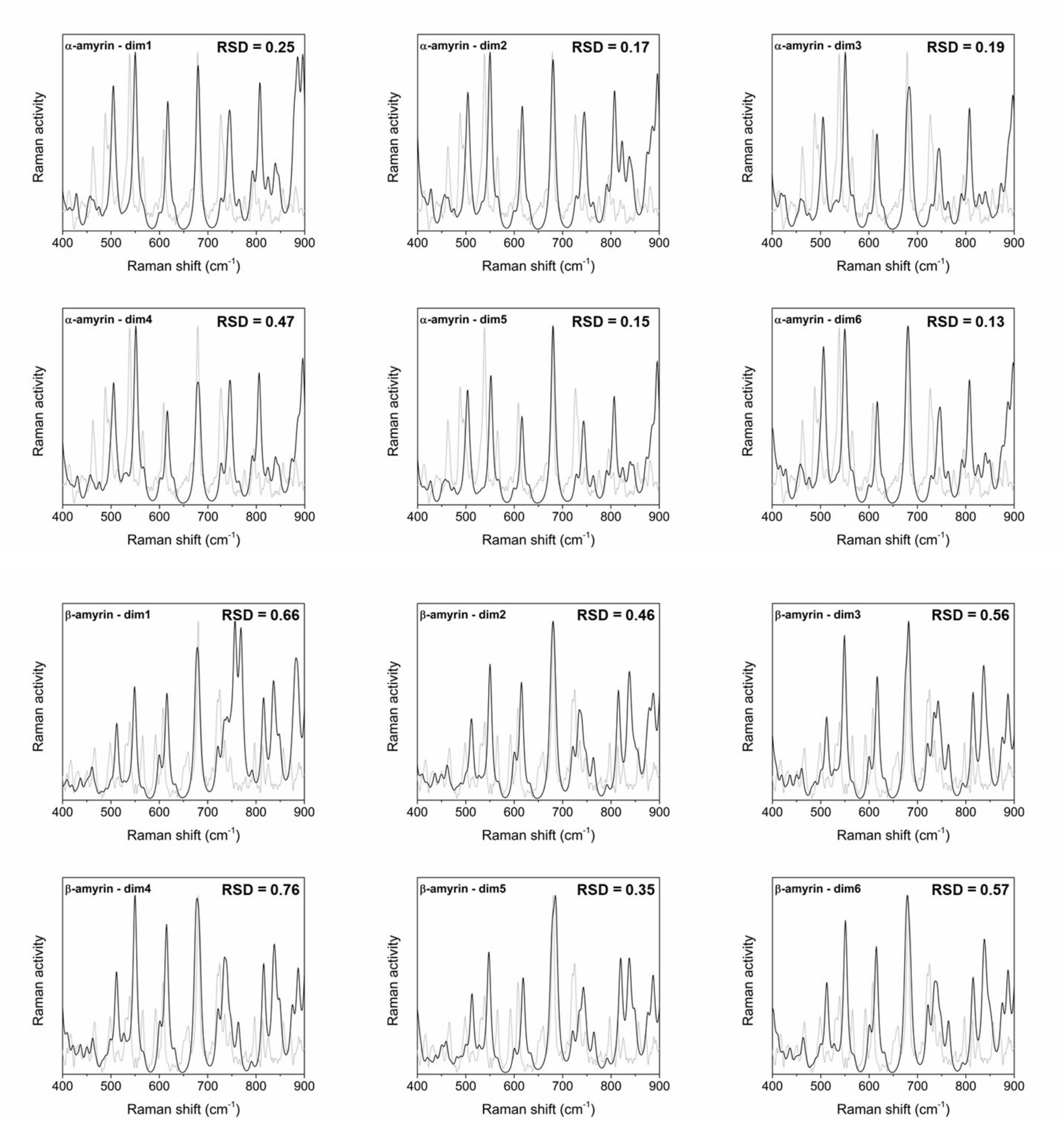

n-OCTANOL

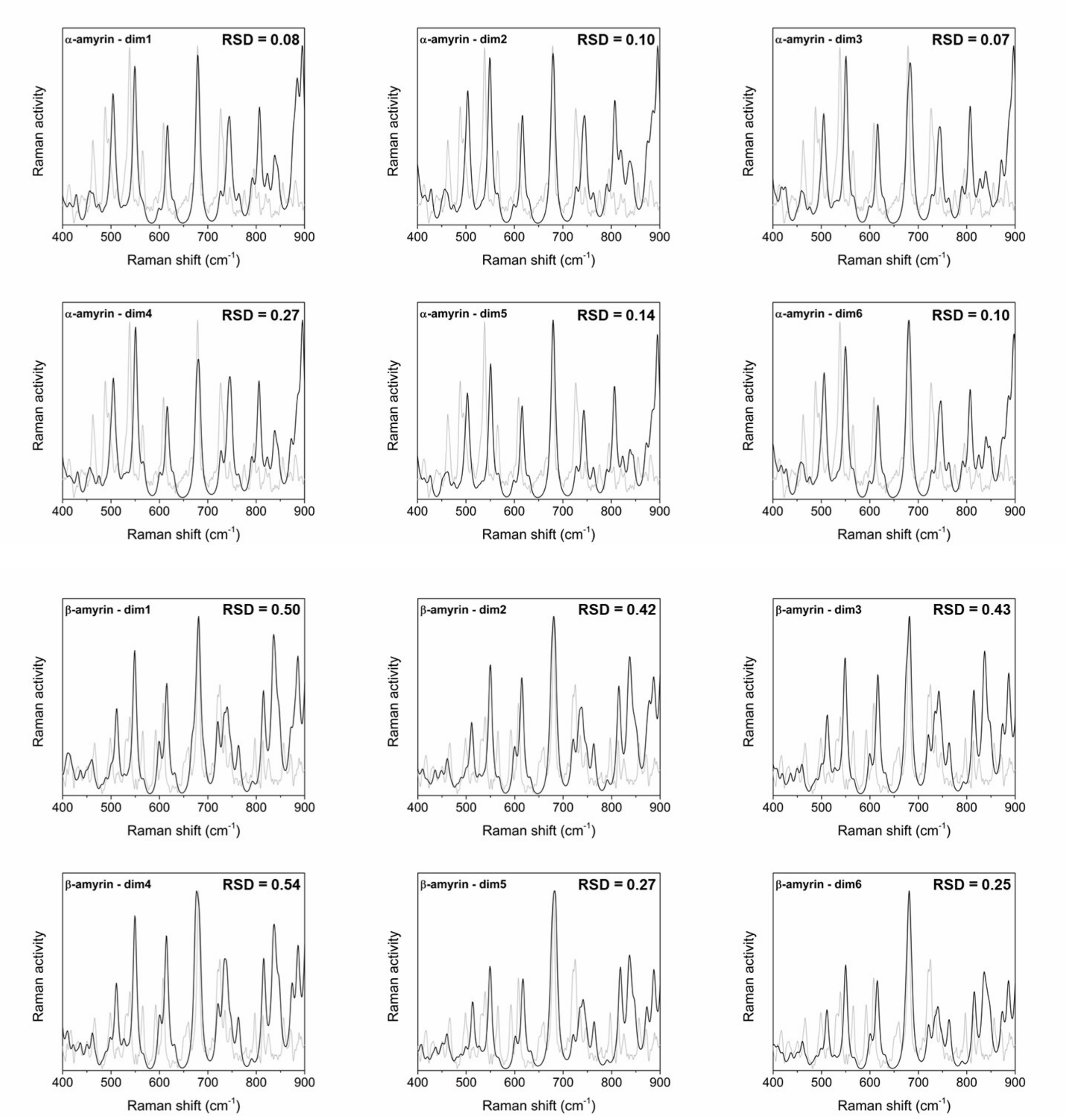

α-amyrin - dim1
RSD = 0.08
α-amyrin - dim2
RSD = 0.10
α-amyrin - dim3
RSD = 0.07
α-amyrin - dim4
RSD = 0.27
α-amyrin - dim5
RSD = 0.14
α-amyrin - dim6
RSD = 0.10
β-amyrin - dim1
RSD = 0.50
β-amyrin - dim2
RSD = 0.42
β-amyrin - dim3
RSD = 0.43
β-amyrin - dim4
RSD = 0.54
β-amyrin - dim5
RSD = 0.27
β-amyrin - dim6
RSD = 0.25
Raman activity
Raman shift (cm-1)
400 500 600 700 800 900

n-HEXANE

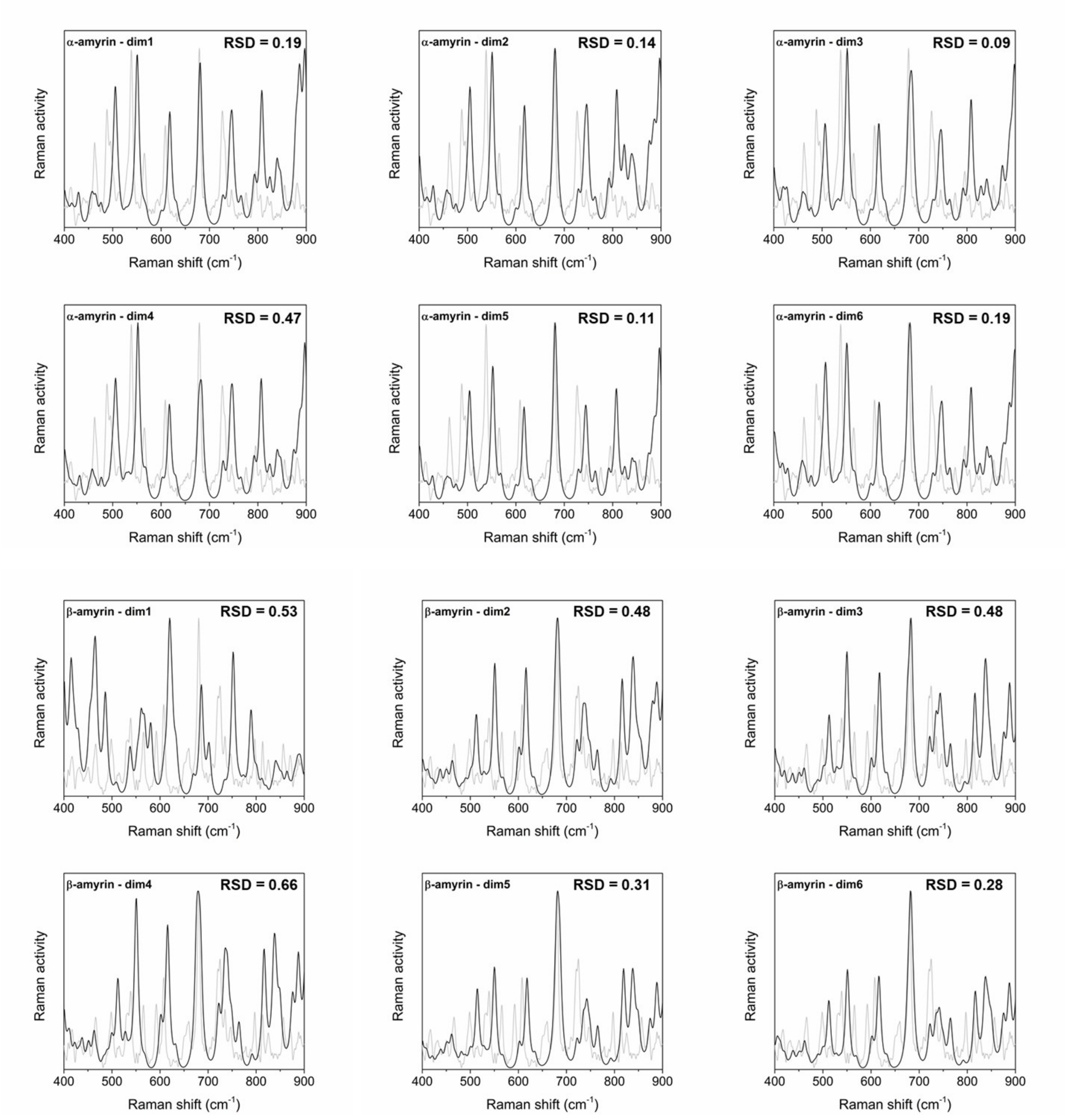
α-amyrin - dim1
RSD = 0.19
α-amyrin - dim2
RSD = 0.14
α-amyrin - dim3
RSD = 0.09
α-amyrin - dim4
RSD = 0.47
α-amyrin - dim5
RSD = 0.11
α-amyrin - dim6
RSD = 0.19
β-amyrin - dim1
RSD = 0.53
β-amyrin - dim2
RSD = 0.48
β-amyrin - dim3
RSD = 0.48
β-amyrin - dim4
RSD = 0.66
β-amyrin - dim5
RSD = 0.31
β-amyrin - dim6
RSD = 0.28
Raman activity
Raman shift (cm-1)
400
500
600
700
800
900

Figure S6.- Relative intensity patterns of the calculated dimeric aggregates for α- and β-amyrin together with the experimental Raman spectra.

NO PCM CORRECTION

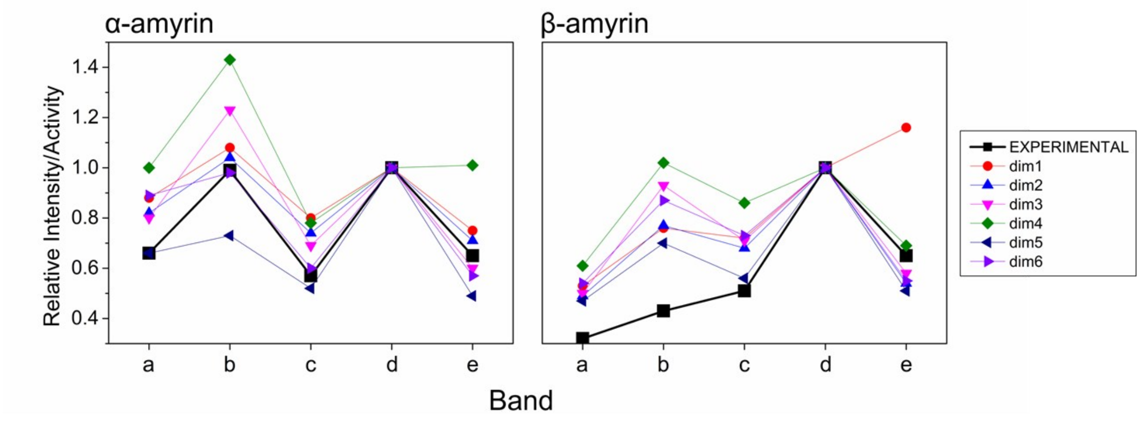

n-OCTANOL

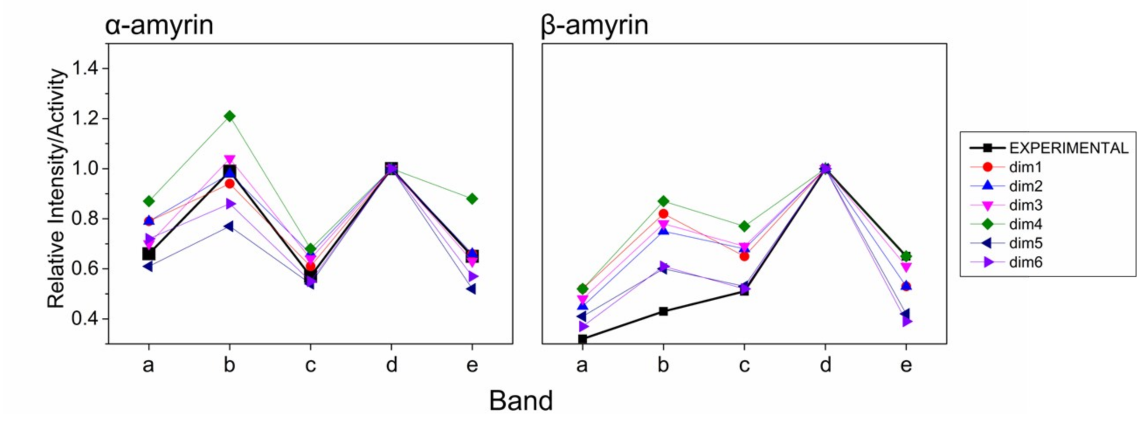

n-HEXANE

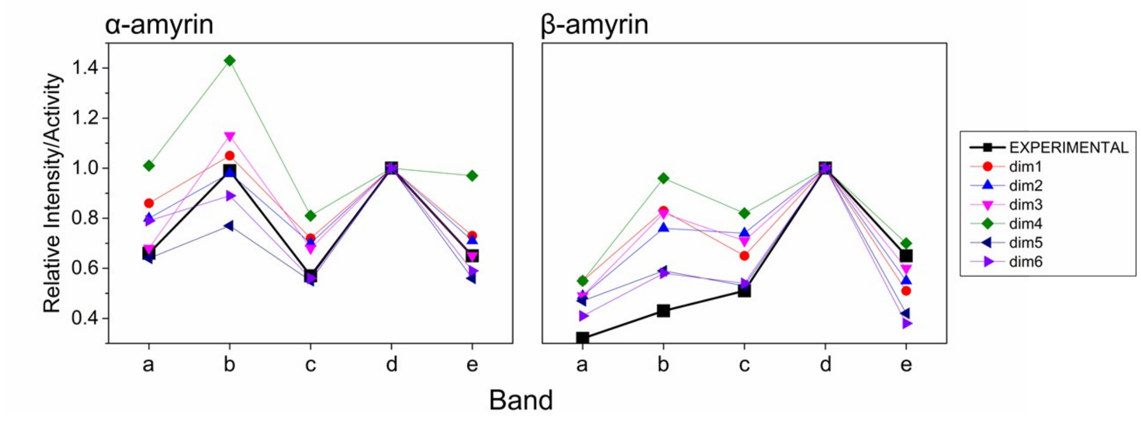

Figure S7.- Experimental intermolecular distances for α- and β-amyrin derivatives obtained from CIF files.

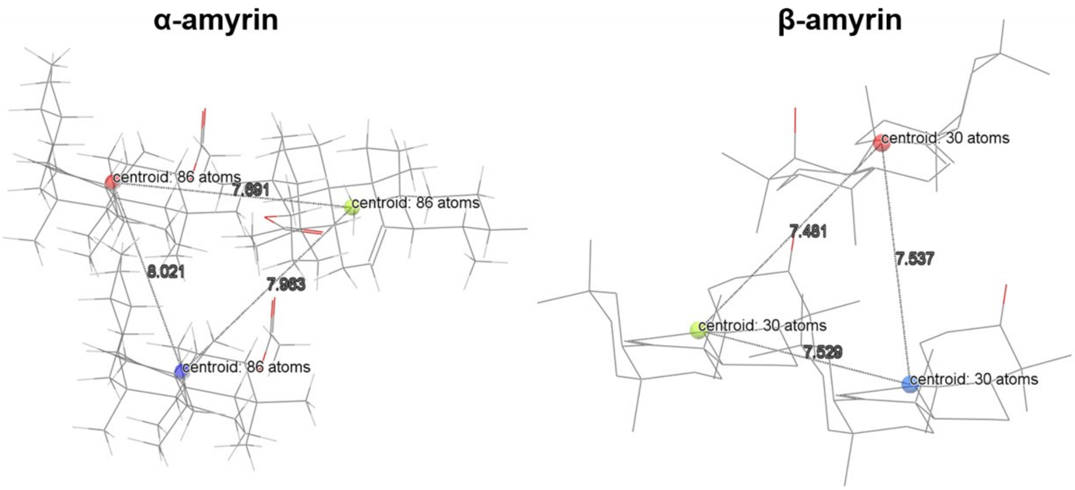

	distances		
**alfa**	7.691	7.963	8.021
**beta**	7.481	7.529	7.537

Figure S8.- Comparison of the experimental Raman spectra at room temperature (gray) with those calculated for α- and β-amyrin trimers in the three PCM options (black).

NO PCM CORRECTION

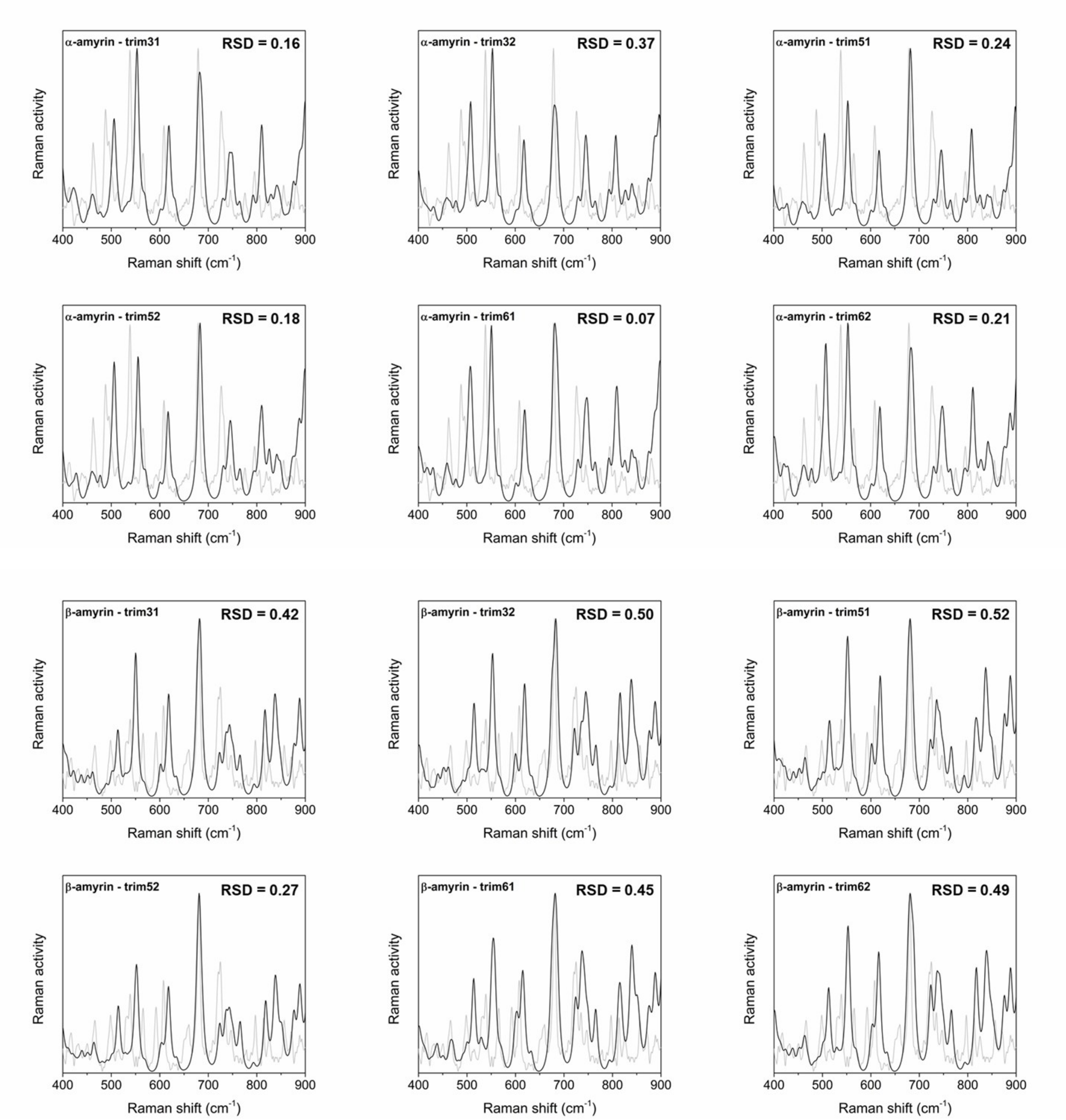

n-OCTANOL

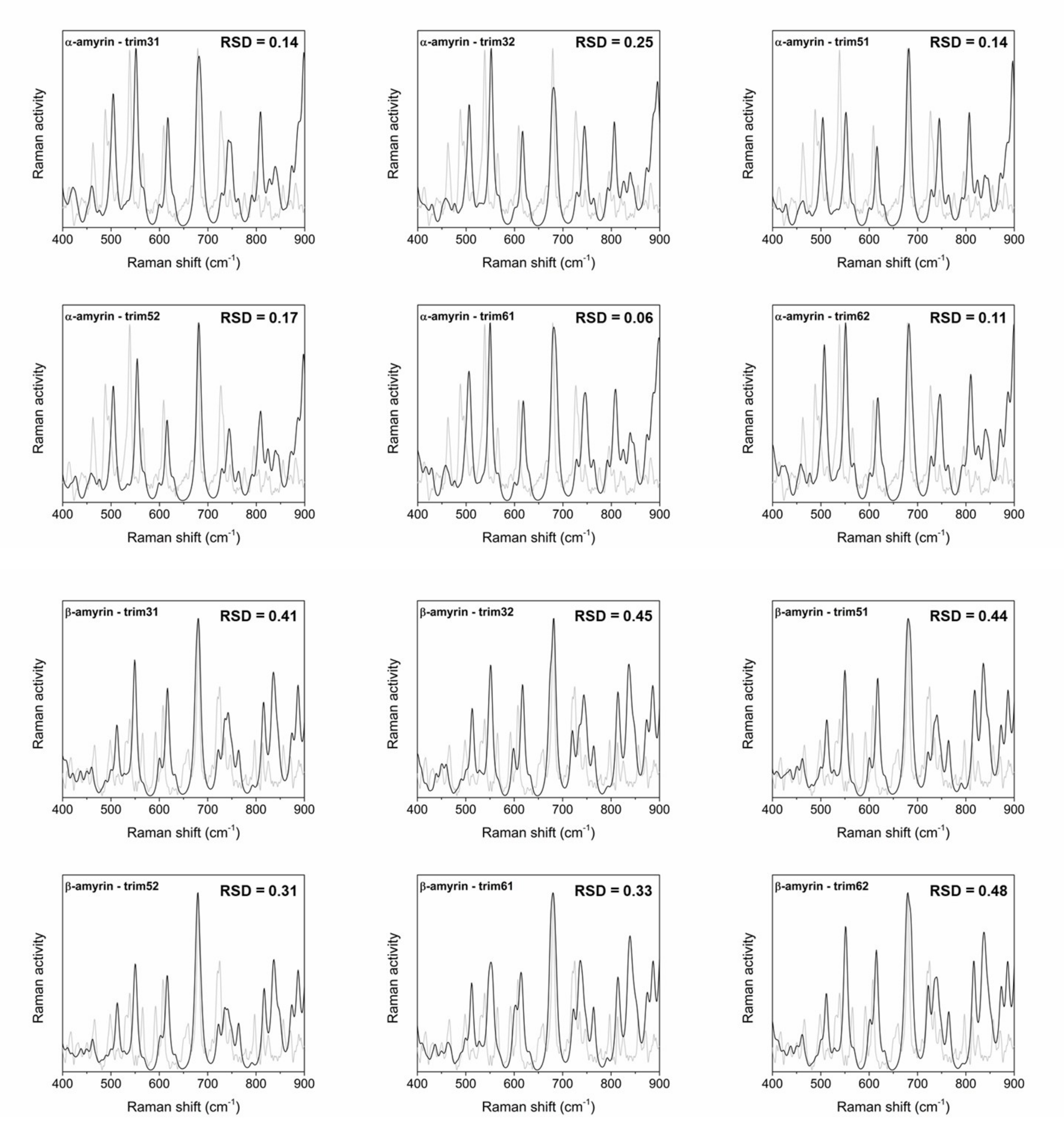
α-amyrin - trim31
RSD = 0.14
α-amyrin - trim32
RSD = 0.25
α-amyrin - trim51
RSD = 0.14
α-amyrin - trim52
RSD = 0.17
α-amyrin - trim61
RSD = 0.06
α-amyrin - trim62
RSD = 0.11
β-amyrin - trim31
RSD = 0.41
β-amyrin - trim32
RSD = 0.45
β-amyrin - trim51
RSD = 0.44
β-amyrin - trim52
RSD = 0.31
β-amyrin - trim61
RSD = 0.33
β-amyrin - trim62
RSD = 0.48
Raman activity
Raman shift (cm-1)
400 500 600 700 800 900

***n*-HEXANE**

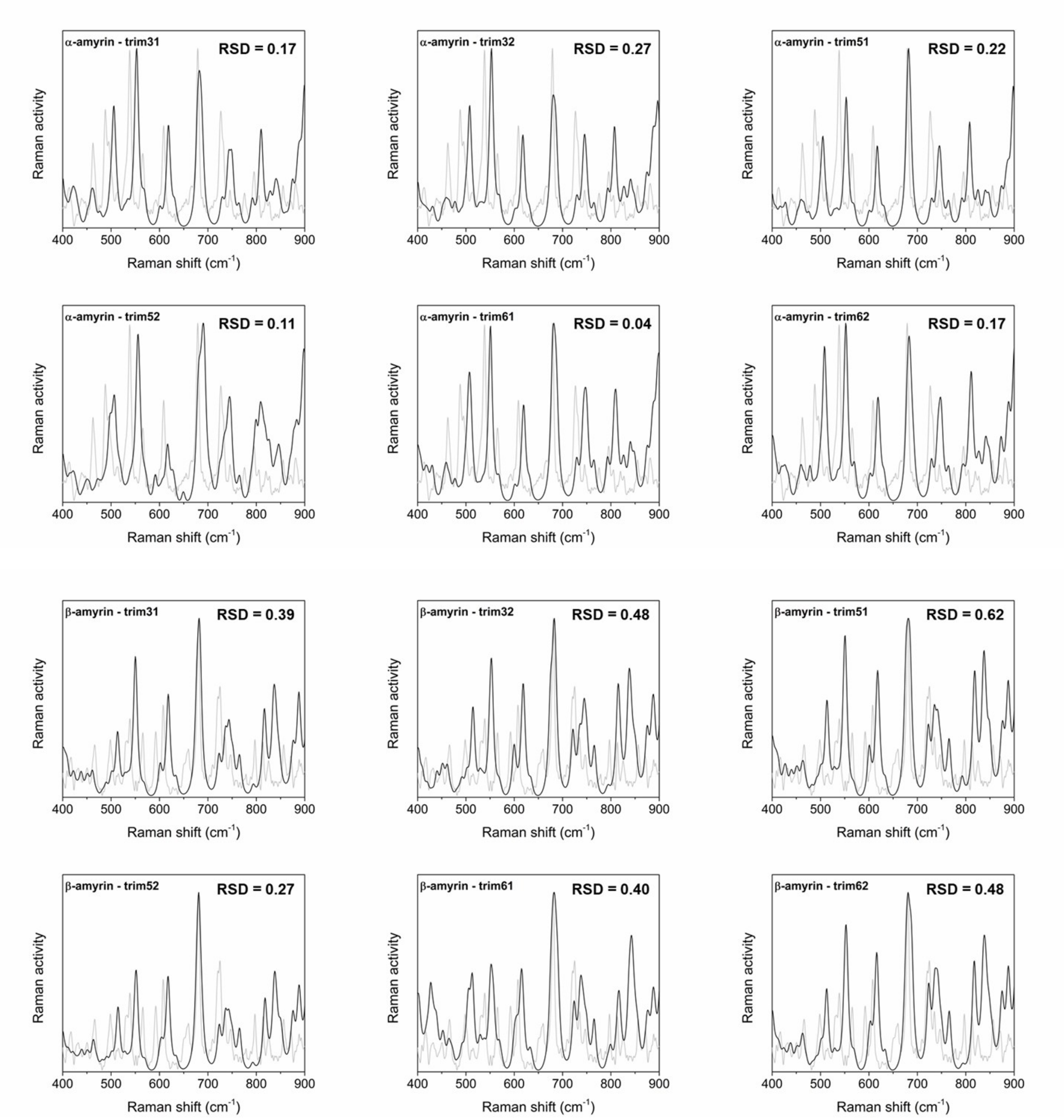

α-amyrin - trim31
RSD = 0.17
α-amyrin - trim32
RSD = 0.27
α-amyrin - trim51
RSD = 0.22
α-amyrin - trim52
RSD = 0.11
α-amyrin - trim61
RSD = 0.04
α-amyrin - trim62
RSD = 0.17
β-amyrin - trim31
RSD = 0.39
β-amyrin - trim32
RSD = 0.48
β-amyrin - trim51
RSD = 0.62
β-amyrin - trim52
RSD = 0.27
β-amyrin - trim61
RSD = 0.40
β-amyrin - trim62
RSD = 0.48
Raman activity
Raman shift (cm-1)
400
500
600
700
800
900

Figure S9.- Relative intensity patterns of the calculated trimeric aggregates in *n*-octanol for α- and β-amyrin together with the experimental Raman spectra.

NO PCM CORRECTION

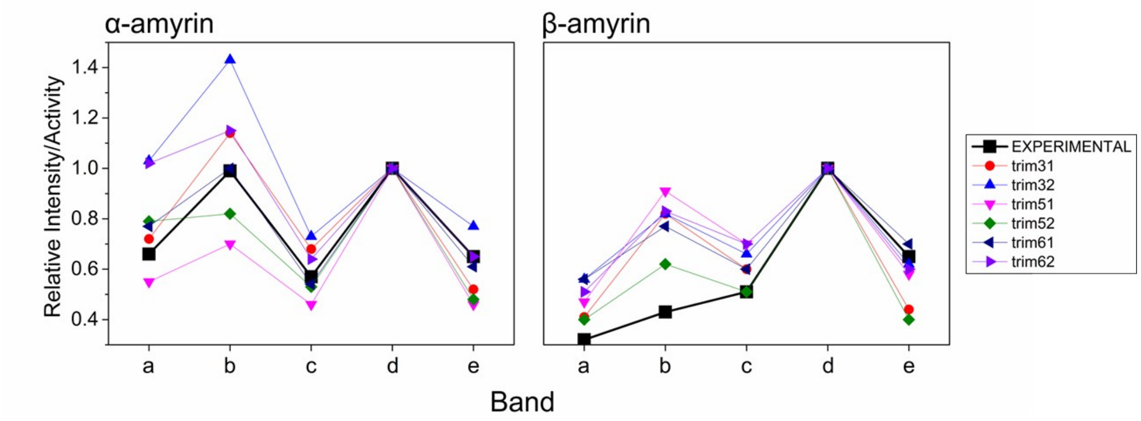

n-OCTANOL

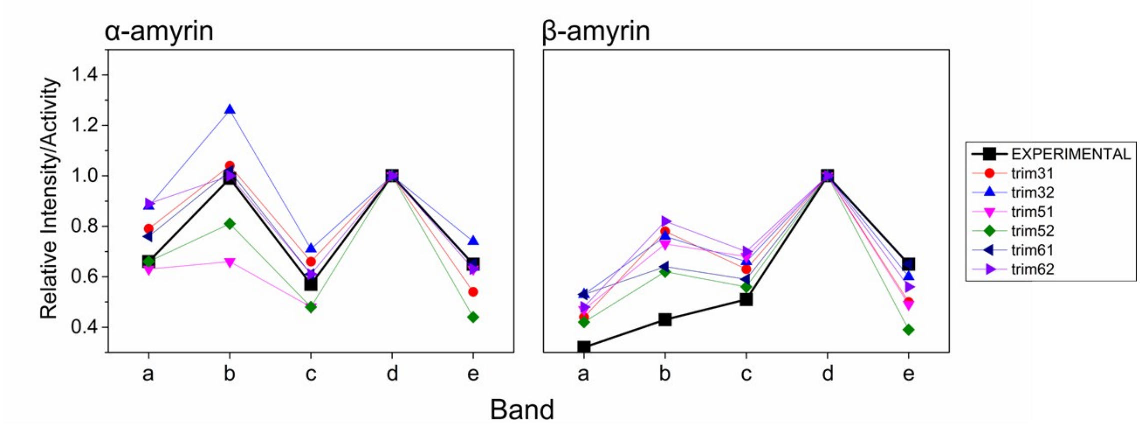

n-HEXANE

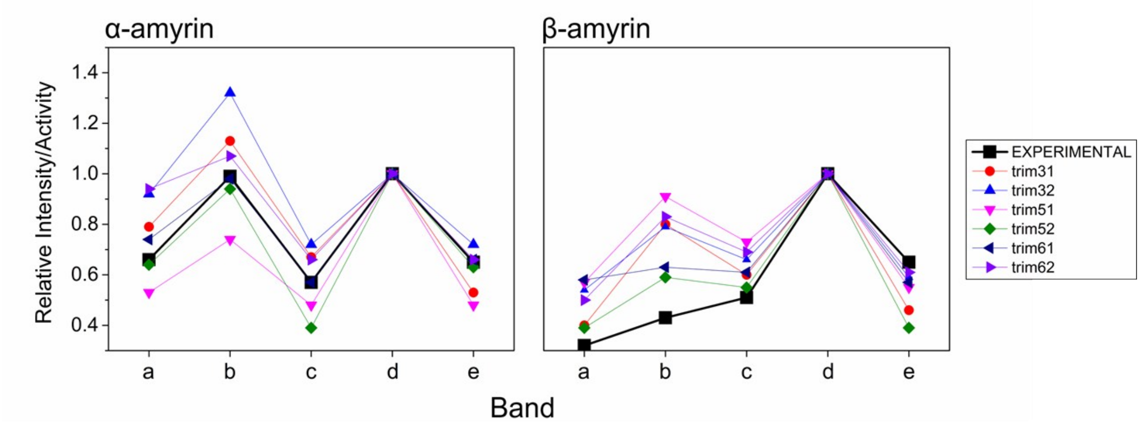

Figure S10.- Relative concentrations of amyrin conformers obtained by Maxwell–Boltzmann populations in dimer M-B and trimer M-B.

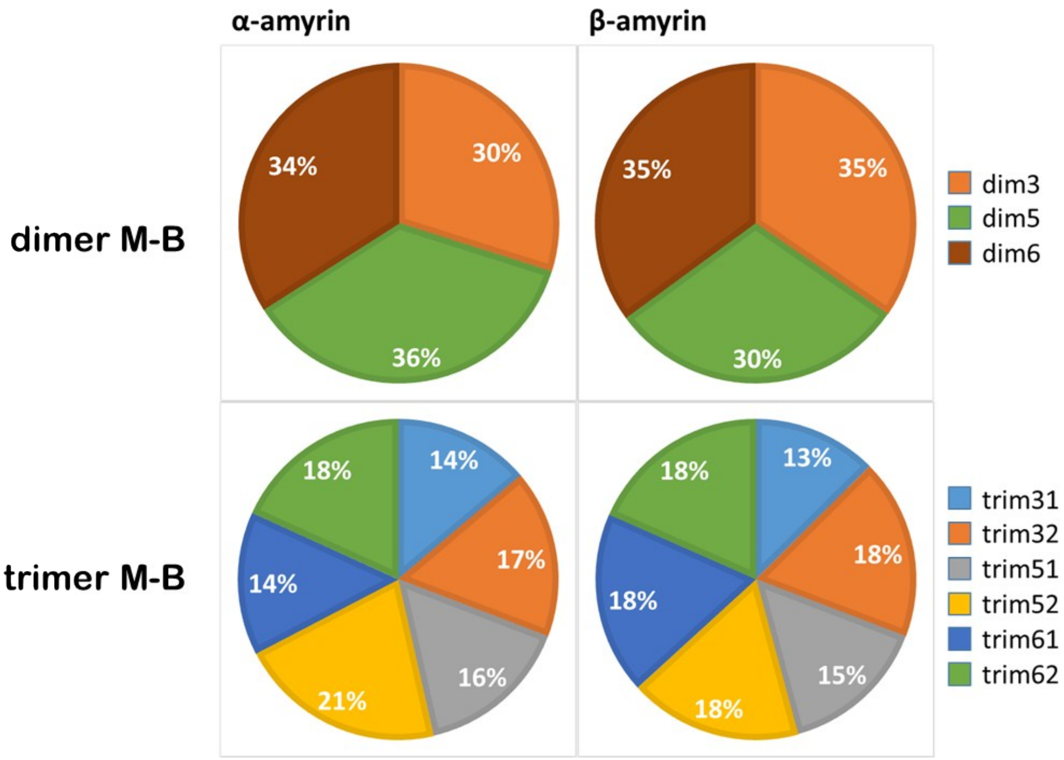

Table TS5.- Relative standard deviation (RSD) values for the calculated Raman spectra in *n*-octanol compared with the experimental ones obtained at different temperatures. Results are shown for molecular stacking aggregates (dimers and trimers) and their Maxwell-Boltzmann distributed average (M-B).

	**α-amyrin**				**β-amyrin**			
**Aggregation**	**300K**	**200K**	**150K**	**77K**	**300K**	**200K**	**150K**	**77K**
**dim3**	0.07	0.08	0.13	0.13	0.43	0.33	0.36	0.32
**dim5**	0.14	0.12	0.09	0.08	0.27	0.19	0.21	0.18
**dim6**	0.10	0.10	0.09	0.08	0.25	0.18	0.19	0.17
**M-B dimers**	0.09	0.08	0.06	0.05	0.26	0.18	0.20	0.17
**trim31**	0.14	0.16	0.20	0.21	0.41	0.32	0.34	0.30
**trim32**	0.25	0.27	0.34	0.33	0.45	0.35	0.38	0.34
**trim51**	0.14	0.13	0.13	0.11	0.44	0.34	0.37	0.33
**trim52**	0.17	0.17	0.16	0.15	0.31	0.24	0.26	0.22
**trim61**	0.06	0.08	0.14	0.13	0.33	0.27	0.30	0.24
**trim62**	0.11	0.13	0.18	0.18	0.48	0.38	0.40	0.36
**M-B trimers**	0.11	0.11	0.10	0.09	0.35	0.26	0.28	0.25
